# 793. Procalcitonin-guided Antibiotic Stewardship Oversight Reduces Antibiotic Use and Adverse Outcomes for Patients Admitted with Pneumonia, Results from a Real-world Randomized Controlled Trial in US Hospitals

**DOI:** 10.1093/ofid/ofac492.054

**Published:** 2022-12-15

**Authors:** Michael Mansour

**Affiliations:** Massachusetts General Hospital, Boston, Massachusetts

## Abstract

**Background:**

ProSAVE (NCT04158804) is the first prospective interventional RCT to address the effect of procalcitonin (PCT)-guidance in the setting of antimicrobial stewardship (ASP) oversight on clinical outcomes and antibiotics usage for patients admitted with pneumonia.

**Methods:**

Analysis of the pilot cohort from a multicenter trial was conducted enrolling subjects admitted with pneumonia at three clinical sites (academic and community hospitals). Subjects meeting eligibility criteria were randomized 1:1 between the standard of care (SoC) versus PCT-guided ASP oversight (PCT arm); subjects were monitored daily by chart review for up to 30 days. An FDA-approved PCT algorithm was used to guide antimicrobial usage.

95 patients were enrolled as ITT of which 60 patients were assigned per-protocol who were adherent to the PCT algorithm (30 in SoC and 30 in the PCT arm). Primary study endpoints were antimicrobial usage and composite safety comprising readmission, death, intubation, septic shock, renal failure, and pneumonia complications such as empyema and abscess up to day 30.

**Results:**

Baseline subject characteristics were similar between both arms; median age 73y (IQR: 62–86, heart disease (43% SoC vs. 40% PCT arm), and diabetes (23% SoC vs. 29% PCT arm). Early antimicrobial cessation by day 4 of hospitalization was achieved more often in the PCT arm as compared to SoC (ITT: SoC n=5, 11% vs. PCT arm n=14, 29%, p=0.045). In addition, antimicrobial prescription at discharge was lower in the intervention arm (ITT: SoC n=24, 51% vs. PCT arm n=15, 31%, p=0.08; similar and statistically significant efficacy for PP) (Fig 1, panel A). A substantial statistically significant improvement of safety was observed in the PCT arm compared to SoC (reduction by 18.3% (95%-CI: 2.3% – 36.3%) from 23.3% (11.9% – 41.1%, SoC) to 3.3% (0.8% – 16.7%, PCT arm) in PP, similar in ITT) (Fig 1, panel B). Readmission was the main driver for fewer safety events in the intervention arm (SoC 50% vs. PCT arm 33%).

**Conclusion:**

In this pilot cohort of ProSAVE, the introduction of PCT-guided, ASP intervention to medical teams proved to be a successful aid for resourceful antibiotic management decisions resulting in shorter antibiotic durations and significantly lower adverse event rates.

**Disclosures:**

**Michael Mansour, MD, PhD**, ThermoFisher Scientific: Grant/Research Support.

